# Spatial and temporal variations in salt marsh microorganisms of the Wadden Sea

**DOI:** 10.1002/ece3.8767

**Published:** 2022-03-27

**Authors:** Maria Rinke, Mark Maraun, Stefan Scheu

**Affiliations:** ^1^ J.F. Blumenbach Institute of Zoology and Anthropology, Animal Ecology University of Göttingen Göttingen Germany; ^2^ Centre of Biodiversity and Sustainable Land Use University of Göttingen Göttingen Germany

**Keywords:** coast, flooding, North Atlantic, phospholipid fatty acid analysis, PLFA, seasonality, spatial dynamics

## Abstract

Salt marshes exist at the interface of the marine and the terrestrial system. Shore height differences and associated variations in inundation frequency result in altered abiotic conditions, plant communities, and resource input into the belowground system. These factors result in three unique zones, the upper salt marsh (USM), the lower salt marsh (LSM), and the pioneer zone (PZ). Marine detritus, such as micro‐ and macroalgae, is typically flushed into the PZ daily, with storm surges moving both salt marsh detritus and marine detritus into higher salt marsh zones. Microbial assemblages are essential for the decomposition of organic matter and have been shown to sensitively respond to changes in abiotic conditions such as oxygen supply and salinity. However, temporal and spatial dynamics of microbial communities of Wadden Sea salt marshes received little attention. We investigated the dynamics of soil microbial communities across horizontal (USM, LSM, and PZ), vertical (0–5 and 5–10‐cm sediment depth), and temporal (spring, summer, and autumn) scales in the Wadden Sea salt marsh of the European North Atlantic coast using phospholipid fatty acid (PLFA) analysis. Our results show strong spatial dynamics both among salt marsh zones and between sediment depths, but temporal dynamics to be only minor. Despite varying in space and time, PLFA markers indicated that bacteria generally were the dominant microbial group across salt marsh zones and seasons, however, their dominance was most pronounced in the USM, whereas fungal biomass peaked in the LSM and algal biomass in the PZ. Only algal markers and the stress marker monounsaturated to saturated fatty acid ratio responded to seasonality. Overall, therefore, the results indicate remarkable temporal stability of salt marsh microbial communities despite strong variability in abiotic factors.

## Graphical abstract

We investigated salt marsh soil microbial community dynamics across spatial scales—shore height and soil depth—as well as temporal scales (spring, summer, and autumn). Spatial dynamics were the strongest factor affecting microbial assemblages, with little evidence of temporal dynamics. Spatial dynamics were related to changes in inundation frequency as a result of shore height.

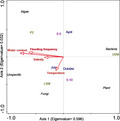

## 1 INTRODUCTION

Salt marshes predominantly form along the interface between the marine and terrestrial system, where erosion by seawater is reduced and sediment gradually accumulates (Adam, 1993; Allen, 2000; Pennings & Bertness, 2001). With the establishment of vascular plants, the sediment is stabilized and continued external addition increases shore height (Adam, 1993; Allen, 2000). Wadden Sea salt marshes are essential in protecting mudflats and the coastline from erosion by the North Sea (Reed et al., 2018; Wang et al., 2012). Furthermore, they sequester large amounts of carbon, thereby functioning as blue carbon systems (McLeod et al., 2011; Mueller et al., 2019). Due to the gradual build‐up, inundation frequency declines with shore height (Bockelmann et al., 2002; Caçador et al., 2007; Roozen & Westhoff, 1985). Changes in inundation frequency result in separate habitats driven by abiotic stressors, such as salinity, water logging, and soil anoxia, as well as competition (Bockelmann et al., 2002; Buth, 1987; Pennings & Bertness, 2001). Salt marshes of the Wadden Sea are split into three separate zones distinguished by their vegetation: the upper salt marsh (USM), the lower salt marsh (LSM), and the pioneer zone (PZ) (Haynert et al., 2017; Winter et al., 2018).

The functioning of salt marshes as carbon sinks results from slow decomposition of highly productive, lignocellulose‐rich terrestrial plants by microbial communities (Hemminga & Buth, 1991; Hemminga et al., 1988; Leadbeater et al., 2021). In addition, inundation of the salt marsh brings allochthonous marine material, such as algal wrack and microphytobenthos, to the marsh adding to the autochthonous organic matter input to the system (Polis & Hurd, 1996; Redzuan & Underwood, 2020; Zong & Horton, 1998). Recent studies indicated the use of these marine resources by soil mesofauna of the salt marsh (Haynert et al., 2017; Winter et al., 2018). The activity of microbes breaking down plant material is influenced by abiotic factors such as soil temperature, water concentration, nutrient addition, and soil anoxia (Hanley et al., 2021; Hemminga & Buth, 1991). Therefore, changes in abiotic pressures across marsh zones may influence microbial processes via changes in microbial community composition and thereby their functioning as blue carbon system. However, most studies focusing on the breakdown of detrital material and microbial activity, investigated North American salt marshes (Gandy & Yoch, 1988; Hanley et al., 2021; Newell et al., 1989; Sherr & Payne, 1978), differing from Wadden Sea marshes in their microtidal range (<2 m) and their organogenic nature resulting in the accretion of organic material, rather than sediment (Allen, 2000; Kearney & Turner, 2016). Studies on European marshes focused on the rate of decomposition, the contribution of fungi or bacteria, and the fate of detrital material of salt marsh plants (Benner et al., 1984; Bouchard et al., 1998; Bouchard & Lefeuvre, 2000). In addition, the importance of benthic microphytobenthos for benthic macroinvertebrates and bacteria of the Wadden Sea mudflats has been extensively studied (Christianen et al., 2017; Middelburg et al., 2000; Taylor et al., 2013). Until today, however, the structure of microbial communities within European salt marshes has not been investigated in detail.

Soil microbial communities exist at the base of the food web and are key in the breakdown of organic matter and nutrient cycling (Bardgett et al., 1999; Buckley & Schmidt, 2001; Gray et al., 2011; Vestal & White, 1989). Microbial communities include bacteria, archaea, protozoans, fungi, micro‐metazoans, and algae (Vestal & White, 1989). The composition of these communities determines the rate of substrate utilization, and also the type of organic and inorganic material used as substrate (Gray et al., 2011). For example, Gram‐positive bacteria may break down recalcitrant materials, whereas Gram‐negative bacteria rely on labile root exudates (Fanin et al., 2019). Studies have demonstrated the sensitivity of microbial communities to external factors such as temperature, precipitation, pH, and atmospheric CO₂ concentrations (Alzarhani et al., 2019; Buckley & Schmidt, 2001; Gray et al., 2011). In salt marshes of Europe, research thus far has focused on the decomposition of residues of specific vascular plants, such as *Spartina alterniflora*, *S. anglica*, and *Atriplex portulacoides* (Benner et al., 1984; Bouchard et al., 1998; Buchan et al., 2003; Hemminga & Buth, 1991; Hemminga et al., 1988), the species and biomass of bacteria and fungi (Buchan et al., 2003; Carvalho et al., 2004; Castro & Freitas, 2000; Leadbeater et al., 2021) as well as which group predominantly breaks down lignocellulose (Benner et al., 1984; Calado et al., 2019; Cortes‐Tolalpa et al., 2018; Leadbeater et al., 2021). In contrast, the overall microbial community composition and their dynamics over time and zonation in the salt marsh received little attention. Therefore, knowledge of these communities is still limited, mainly due to the vast numbers of microbial species and difficulties in cultivating and quantifying them (Buckley & Schmidt, 2001; Vestal & White, 1989). Recently, DNA metabarcoding has been used to identify spatial dynamics influencing benthic diatom communities in salt marshes (Plante et al., 2021). However, lipid analysis, more specifically phospholipids, allowing the quantification of microbial biomass, community composition, substrate availability, and metabolic status (Bossio & Scow, 1998; Vestal & White, 1989; Zelles, 1999), has not been used for analyzing variations in microbial communities across salt marsh zones.

Phospholipid fatty acid (PLFA) analysis is based on the fact that some fatty acids (FAs) are only produced by certain microorganisms (Vestal & White, 1989; Zelles, 1999). In addition, specific PLFA ratios serve as indicators of physiological stress and nutrient availability (Bossio & Scow, 1998; Pollierer et al., 2015; Wixon & Balser, 2013). Physiological stress in Gram‐negative bacteria is indicated by the ratio of cyclopropyl lipids to their monounsaturated precursors (Gray et al., 2011; Pollierer et al., 2015; Willers et al., 2015). With increased stress, such as temperature rise or lack of oxygen, microorganisms enter a state of reduced growth (Wixon & Balser, 2013) associated with increased conversion of monounsaturated lipids to cyclopropyl lipids (Guckert et al., 1986; Kaur et al., 2005). Furthermore, monounsaturated lipids were shown to increase with greater substrate availability and decrease with flooding intensity (Bossio & Scow, 1998; Pollierer et al., 2015). As a result, the ratio of monounsaturated to saturated precursor lipids is indicative of aerobic activity as well as substrate availability to microorganisms (Bossio & Scow, 1998; Pollierer et al., 2015). Previous research has indicated seasonal changes in microbial PLFA signatures with changing abiotic conditions (Moore‐Kucera & Dick, 2008; Pollierer et al., 2015), confirming that microbial community changes can be assessed across temporal scales. Therefore, phospholipid analysis allows the quantification of functional groups such as fungi, Gram‐positive, and Gram‐negative bacteria as well as their variations with abiotic conditions. Measuring phospholipids across habitats and time allows insight into the dynamics of these functional groups.

The aims of this study were to investigate the structure of microbial communities, the use of allochthonous algal material and physiological stress indicators across the salt marsh soil, and their temporal and spatial changes using PLFAs. More specifically, we hypothesized that (1) the concentration of PLFA markers changes across salt marsh zones, with the algal marker concentration increasing toward lower zones (LSM and PZ), the stress indicator cyclic to precursor FA ratio being highest in the USM due to higher temperatures and more frequent droughts, and the monounsaturated to saturated FA ratio being highest in the USM due to greater availability of oxygen, organic material, and low inundation frequency. Furthermore, we hypothesized that (2) due to changes in abiotic conditions and tidal regime in the marsh, PLFA marker concentration varies across seasons with the cyclic to precursor FA ratio increasing in summer due to increased stress by high temperature and drought, and the monounsaturated to saturated FA ratio increasing due to increased oxygen supply. Finally, we hypothesized (3) the concentration of PLFA markers to decrease with sediment depth due to reduced resource input and redox potential; due to the association of monounsaturated FAs with aerobic growth, we hypothesized the monounsaturated to saturated FA ratio to decrease with sediment depth, whereas the cyclic to precursor FA ratio to increase. Overall, this study is expected to contribute to the identification of the factors driving the temporal and spatial dynamics of microbial communities and carbon sequestration in salt marsh soil.

## 2 METHODS

### 2.1 Sampling

Samples were taken across five transects on the island of Spiekeroog (Wadden Sea National Park, Germany; 53°45′2″–53°47′1″N, 7°40′0″–7°49′1″E) in April (16th, three days prior to spring tide), July (16th, during spring tide), and October (22nd, one day past neap tide) 2019. The three sampling dates were taken to represent three seasons, that is, spring, summer, and autumn, and thereby the temporal variation in microbial communities. Per transect and zone one soil core (ø 5 cm) was taken and separated into two sediment depths, 0–5 and 5–10 cm, resulting in 30 cores per sampling date. The cores were stored at −20°C until further processing. Prior to extraction of PLFAs, each core was sieved (2 mm) and material other than soil, such as organic material and buried animals, was removed.

Samples were taken from the USM, LSM, and PZ. The USM is situated 35 cm above the mean high water level (MHWL) to the storm tide limit, it is inundated between 35 and 70 times a year with a soil salinity between 5 and 20 ‰ and is dominated by *Elymus athericus* (= *Elytrigia atherica*) (Niedringhaus, 2009). The LSM is located 0–35 cm above the MHWL and flooded between 150 and 250 times a year with a soil salinity of 20‰–26‰; it is characterized by *Atriplex portulacoides* and *Puccinellia maritima*. The PZ, situated below the MHWL, is typically inundated twice a day with a soil salinity of 26‰–32‰ and characterized by *Salicornia* sp. and *Spartina anglica* as well as macroalgae such as *Fucus* sp. and *Ulva* sp. (Haynert et al., 2017; Niedringhaus, 2009; Winter et al., 2018).

### 2.2 PLFA analysis

The extraction, lipid separation, and transesterification of lipids followed the protocol of Buyer and Sasser (2012) with minor adjustments. Approximately 2 g of soil was weighed into 10‐ml glass tubes, dried overnight in a vacuum centrifuge at room temperature, and the dry weight determined. For the extraction, a predetermined volume (1000 µl) of the organic phase was evaporated prior to lipid separation.

Lipid separation was performed on a 96‐well plate (Thermo‐Scientific, Silica 96 Well Plate, 50 mg). Once separated, 20 µl of internal standard was added to each sample. Transesterification was done with CHCl₃ and 0.075 M CH₃COOH with 0.7 ml of sample removed and evaporated. Samples were eluted with isooctane into vial inserts, placed into 1.5‐ml vials, capped, and stored at −20°C until gas chromatography. Lipids were separated using a gas chromatograph (Clarus 500, PerkinElmer, Norwalk, USA) equipped with an Elite‐5 capillary column (30 m × 0.32 mm i.d., film thickness 0.25 mm, PerkinElmer, Norwalk, USA). The analysis started with 60°C for 1 min, then increased by 30°C/min to 160°C; followed by 3°C/min increase to 260°C; the injection temperature was 250°C, the carrier gas was helium.

Lipids were identified by retention time based on standard mixtures composed of 37 fatty acid methyl esters (FAMEs; Sigma‐Aldrich, St Louis, USA) ranging between C11 and C24 chain lengths, as well as 26 bacterial acid methyl esters (BAMEs; Sigma‐Aldrich, St Louis, USA), and algal standards for 16:2ω6,9 and 16:3ω3,6,9 (Larodan AB, Solna, Sweden) (Buse et al., 2013).

Fatty acids were allocated to marker groups. Vascular plant markers included 18:1ω9, 22:0, and 24:0 (Ruess & Chamberlain, 2010; Zelles, 1999). Bacterial markers included 15:0, 16:1ω7, 17:0, 18:1ω7, 2‐OH‐12:0, a15:0, cy17:0, cy19:0, i15:0, i16:0, and i17:0 (Haynert et al., 2020; Ruess & Chamberlain, 2010; Zelles, 1999); 18:2ω6,9 was taken as general fungal marker (Frostegard & Baath, 1996). Algal markers included 14:0, 16:2ω6,9, 16:3ω3,6,9, 20:5ω3,6,9,12,15, and 22:6ω3,6,9,12,15,18 (Buse et al., 2013; Kelly & Scheibling, 2012). Changes in the PLFA concentration of the complement of marker groups were taken to represent changes in microbial community composition. To get further insight into microbial community composition and functioning, we calculated three indices. The fungi/bacteria ratio was calculated as 18:2ω6,9 / sum of (i15:0 + a15:0 + 15:0 + i16:0 + 16:1ω7 + i17:0 + cy17:0 + 17:0 + 18:1ω7 + cy19:0) (Frostegard & Baath, 1996; Pollierer et al., 2015; Wixon & Balser, 2013). Furthermore, two stress indicator ratios were included. The cyclic phospholipids to their precursor (cy/pre) ratio, calculated as cy17:0/16:1ω7 (cy19:0 was only present in one sample and therefore omitted) (Bossio & Scow, 1998; Moore‐Kucera & Dick, 2008; Wixon & Balser, 2013) indicating physiological or nutritional stress in Gram‐negative bacteria (Bossio & Scow, 1998; Wixon & Balser, 2013). The monounsaturated to saturated precursor (mono/sat) ratio, calculated as sum of monounsaturated FAs (16:1ω7 + 17:1ω7 + 18:1ω7 + 18:1ω9) / sum of saturated FAs (14:0 + 15:0 + 16:0 + 17:0 + 18:0 + 20:0), indicated aerobic growth and substrate availability for microbes (Bossio & Scow, 1998; Wixon & Balser, 2013).

### 2.3 Statistical analyses

PLFA marker lipids and ratios were analyzed by mixed‐effects models with “Zone” (USM, LSM, and PZ), “Season” (spring, summer, and autumn), and “Depth” (0–5 and 5–10 cm) as fixed factors and “coreID” nested within “Transect” as random factor. The statistical analyses were done in R 4.0.5 (R Core Team, 2021) using packages: “emmeans,” “lme4,” “lmerTest,” “effects,” and “tidyverse.” PLFA marker lipid concentrations (nmol g^−1^ dry weight) were log(x+1) transformed to improve homogeneity of variance, PLFA ratios were logit transformed. Means and standard deviation given in text are based on nontransformed data.

Redundancy analysis (RDA) was used for analyzing the composition of marker lipids (as percentages of total) together with abiotic data including soil salinity (Meier, Thölen, Lohmus, et al., 2020), inundation frequency (counts/month) and inundation duration (calculated from water level data; Meier et al., 2020b), soil water content (D. Meier, submitted to PANGAEA), and temperature (Meier et al., 2020a). Soil parameters were measured as described in Balke et al. (2017). Forward selection was used to determine the most important abiotic factors explaining lipid marker composition.

## 3 RESULTS

### 3.1 Marker PLFAs

**FIGURE 1:**
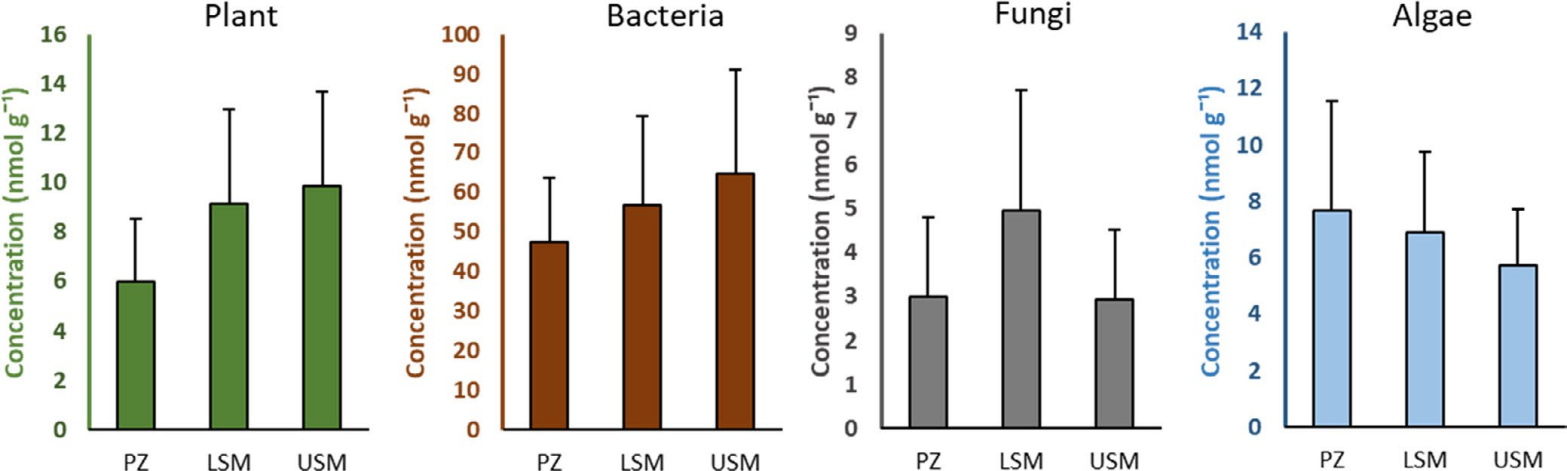
Concentration of marker PLFAs (nmol g⁻¹) for plants (green), bacteria (brown), fungi (gray), and algae (blue) across salt marsh zones (PZ, pioneer zone; LSM, lower salt marsh; USM, upper salt marsh). Error bars show standard deviation. Note different scales of *y*‐axis. Means and standard deviations of original data

Plant marker concentration significantly varied with Depth and Zone, but not with Season (Table 1). Generally, it was much higher in the 0–5‐cm (11.21 ± 3.00 nmol g⁻¹) than in the 5–10‐cm sediment depth (5.48 ± 2.01 nmol g⁻¹). Reduction of the marker with sediment depth was consistent across salt marsh zones and seasons without significant interactions. Among salt marsh zones, plant markers were highest in the USM and LSM, and lower in the PZ (Figure 1).

**TABLE 1:** *F*‐ and *p*‐values of linear mixed‐effects models on variations in PLFA markers (plants, bacteria, fungi, algae) and marker ratios (fungi/bacteria, cy/pre, mono/sat; see Methods for details) with sediment depths (0–5 and 5–10 cm), salt marsh zones (upper salt marsh, lower salt marsh, pioneer zone) and seasons (April, July and October), and their interactions

Marker	Depth (D)		Zone (Z)		Season (S)		D × Z		D × S		Z × S		D × Z × S	
	*F*	*p*	*F*	*p*	*F*	*p*	*F*	*p*	*F*	*p*	*F*	*p*	*F*	*p*
Plant	226.97	**<.001**	39.59	**<.001**	2.30	.107	.35	.704	1.06	.351	1.49	.216	1.50	.212
Bacteria	219.49	**<.001**	13.43	**<.001**	2.93	.060	1.41	.251	.65	.527	1.75	.149	1.02	.403
Fungi	103.02	**<.001**	8.14	.**006**	.32	.726	.77	.466	.52	.597	32.19	.**018**	.95	.441
Algal	208.17	**<.001**	8.05	.**001**	4.22	.**019**	5.29	.**007**	1.18	.314	1.55	.197	2.24	.073
Fungi/bacteria	7.90	.**007**	9.08	.**004**	3.13	.051	.00	.997	.09	.912	6.53	**<.001**	1.36	.260
Cy/Pre	48.89	**<.001**	15.06	.**002**	2.47	.093	1.18	.315	9.36	**<.001**	.53	.718	2.98	.**026**
Mono/Sat	259.18	**<.001**	330.98	**<.001**	34.81	**<.001**	8.33	.**001**	1.88	.161	5.42	.**001**	.56	.693

Note

Significant effects (*p* < .05) are highlighted in bold.

Similar to plant markers, bacterial marker concentration significantly varied with Depth and Zone, with Season being only marginally significant and none of the interactions being significant (Table 1). Generally, in the 0–5‐cm sediment depth (74.91 ± 17.30 nmol g⁻¹) it was about twice as high as in the 5–10‐cm sediment depth (38.95 ± 10.31 nmol g⁻¹). Furthermore, it declined in from the USM to the LSM to the PZ (Figure 1). Of the individual bacterial PLFAs in the upper sediment depth the concentration of 18:1ω7 was greatest (29.26 ± 9.66 nmol g⁻¹), followed by 16:1ω7 (17.02 ± 4.30 nmol g⁻¹), i15:0 (10.82 ± 2.73 nmol g⁻¹), and a15:0 (6.55 ± 1.21 nmol g⁻¹). In the deeper sediment, the concentration of 18:1ω7 also was greatest (12.11 ± 4.73 nmol g⁻¹), followed by i15:0 (7.36 ± 1.95 nmol g⁻¹), 16:1ω7 (7.30 ± 2.45 nmol g⁻¹), and a15:0 (4.65 ± 1.11 nmol g⁻¹).

**FIGURE 2:**
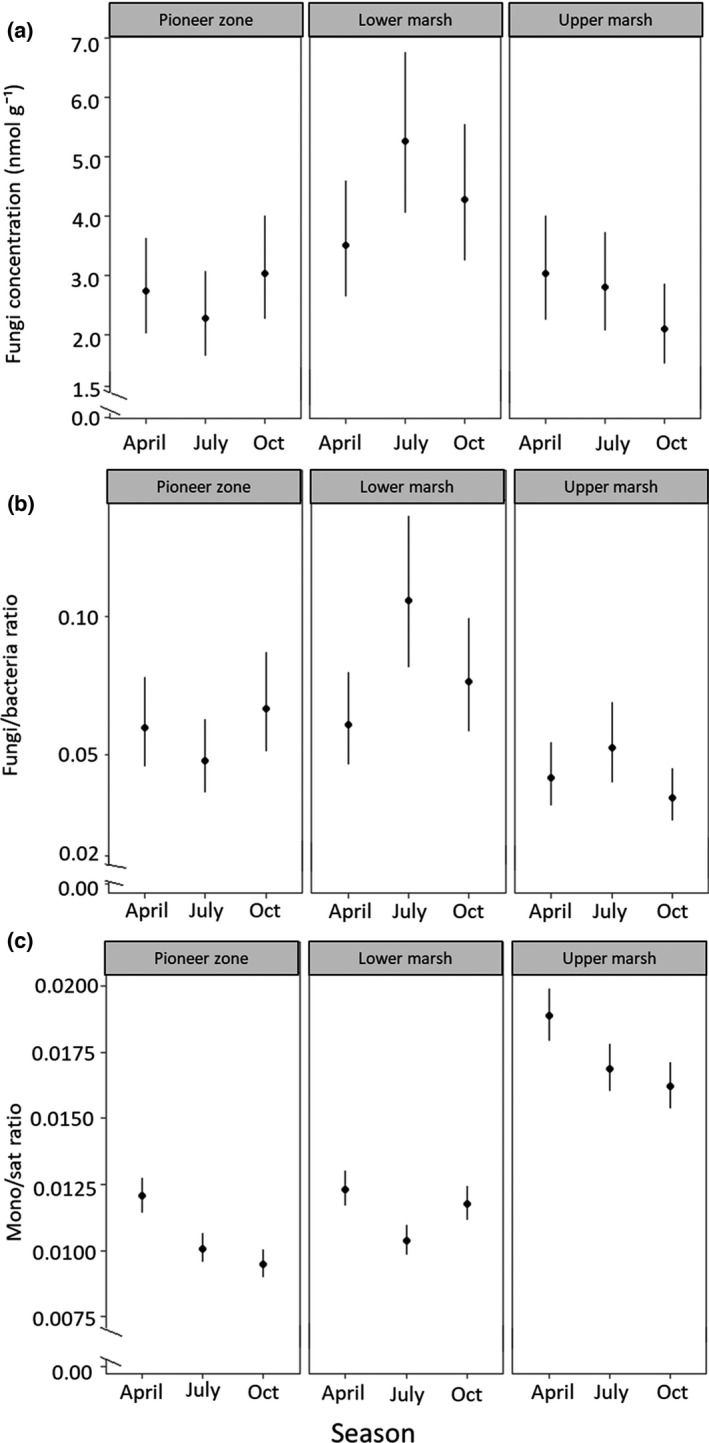
Changes in (a) the fungal marker concentration, (b) the fungi/bacteria PLFA ratio, and (c) monounsaturated/saturated PLFA ratio (both logit transformed) across salt marsh zones and season (April, July, October); estimated means with 95% confidence intervals

Again as for the plant and bacterial markers, the fungal marker 18:2ω6,9 varied significantly with Depth and Zone, but not with Season as main factor, however, in contrast to the plant and bacterial markers, there was a significant interaction between Zone and Season (Table 1). Concentration of the marker was highest in the 0–5‐cm sediment depth (4.96 ± 2.04 nmol g^−1^) and dropped to less than half in the 5–10‐cm sediment depth (2.30 ± 1.71 nmol g^−1^). On average, the fungal marker concentration was similarly low in the USM and PZ, but higher in the LSM (Figure 1). However, in the USM fungal marker concentrations were highest in spring and gradually declined toward autumn, whereas in the LSM it was highest in summer and lower in spring and autumn; in the PZ changes were inverse to those in the LSM (Figure 2a).

**FIGURE 3:**
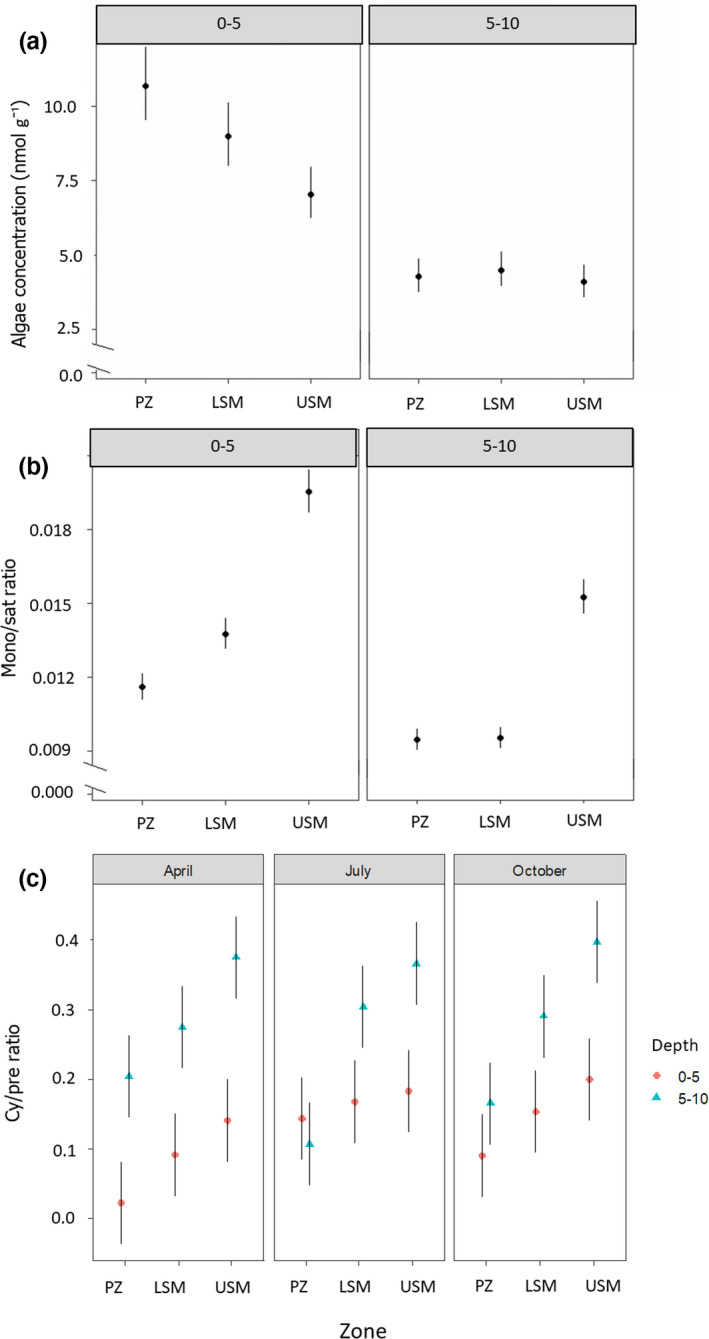
Changes in (a) algae marker concentration with sediment depths across salt marsh zones, (b) the monounsaturated/saturated PLFA ratio with sediment depths across salt marsh zones, and (c) the cyclic/precursor PLFA ratio with sediment depths (0–5 and 5–10 cm) across salt marsh zones (PZ, pioneer zone; LSM, lower salt marsh; USM, upper salt marsh), and seasons (April, July, October); estimated means with 95% confidence intervals

Algal marker concentrations varied significantly across all factors, with a significant interaction between Depth and Zone (Table 1). Concentrations were higher in the 0–5‐cm than in the 5–10‐cm sediment depth, but the difference between the depths was most pronounced in the PZ with concentrations in the 5–10‐cm sediment depth 60% less than the 0–5‐cm. In the LSM, algal marker concentrations halved in 5–10 cm and reduced by 40% in in the USM (Figure 3a). Generally, however, the concentration of the algal marker was similar in the PZ and LSM, but lower in the USM (Figure 1). Across seasons, the algal marker concentration was highest in April (7.54 ± 3.56 nmol g^−1^), and at a similarly low level in July (6.33 ± 2.95 nmol g⁻¹) and October (6.43 ± 2.66 nmol g^−1^). However, individual marker concentrations declined in parallel in the 0–5‐ and 5–10‐cm sediment depths in the order 14:0 (4.18 ± 1.00 and 2.41 ± 0.70 nmol g⁻¹, respectively) > 16:3ω3,6,9 (3.15 ± 0.89 and 1.94 ± 0.81 nmol g⁻¹) > 20:5ω3 (1.69 ± 1.76 nmol g⁻¹ and 0.06 ± 0.27 nmol g⁻¹) > 22:6ω3 (0.09 ± 0.22 nmol g⁻¹ and absent in 5–10 cm). The flagellate marker 22:6ω3 was only found in the 0–5‐cm sediment depth of the PZ in April and July. The diatom marker (20:5ω3,6,9,12,15) concentration was highest in the 0–5‐cm sediment depth of the PZ (3.48 ± 1.79 nmol g⁻¹), followed by the 0–5‐cm sediment depth of the LSM (1.06 ± 0.99 nmol g⁻¹) and the 0–5‐cm sediment depth of the USM (0.53 ± 0.35 nmol g⁻¹); it was very low in the 5–10‐cm sediment depth (0.06 ± 0.27 nmol g⁻¹).

**FIGURE 4:**
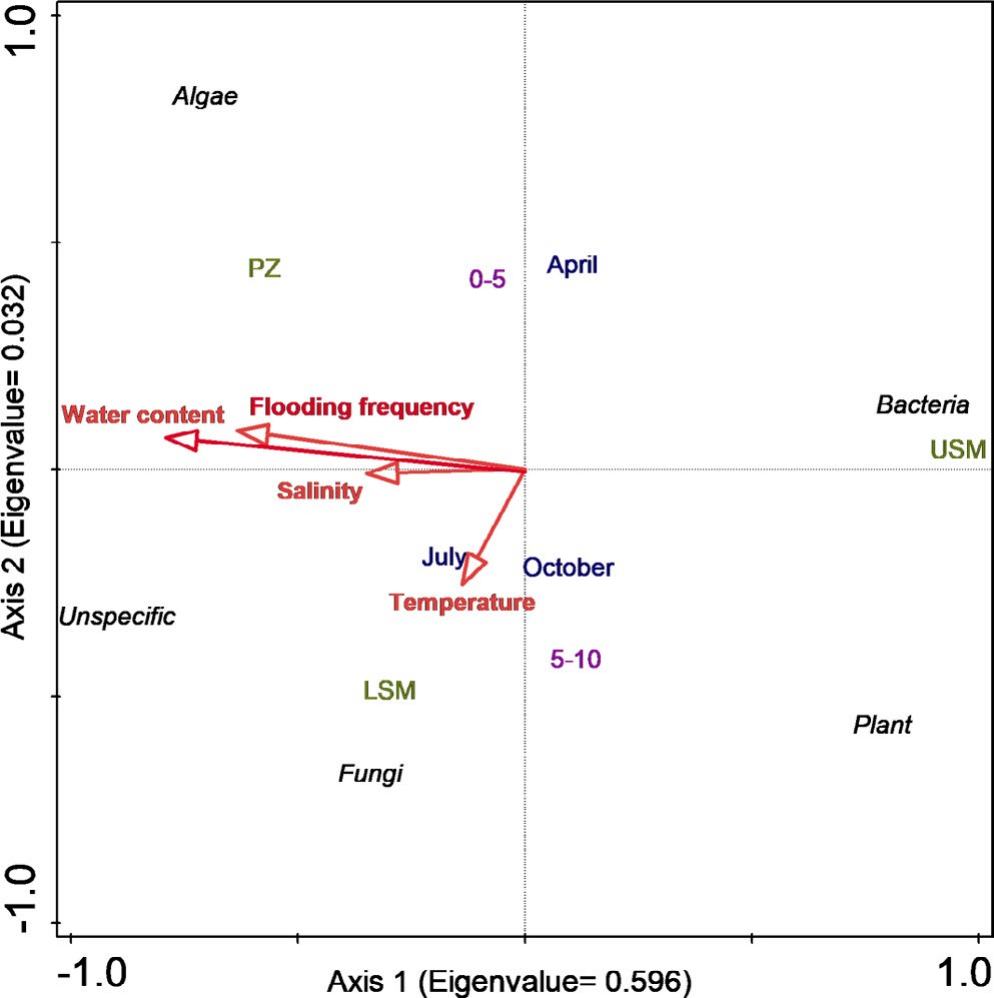
Redundancy analysis of PLFA marker groups with water content, inundation frequency, salinity (‰), and temperature (°C) as explanatory environmental factors. Salt marsh zones (PZ, pioneer zone; LSM, lower salt marsh; USM, upper salt marsh), sediment depth (0–5 and 5–10 cm), and seasons (April, July, October) were included as silent variables not affecting the ordination

Redundancy analysis (RDA) of the marker fatty acids confirmed changes in the concentration of PLFA markers across the salt marsh zones, seasons, and depths (Figure 4). Forward selection indicated the strongest abiotic factors to be water content, salinity, flooding frequency, and soil temperature. The first axis explained 59.6% of the variation and separated the USM from the PZ and LSM, with the separation being mainly due to higher water content (pseudo‐*F* = 82.8, *p* = .002, contribution = 70.8%), soil salinity (pseudo‐*F* = 18.5, *p* = .002, contribution = 13.2%) and flooding frequency (pseudo‐*F* = 10.1, *p* = .002, contribution = 6.2%) in the LSM and PZ. Bacterial and plant marker fatty acids were closely associated with the USM, whereas algal markers were closely associated with the PZ, and the fungal marker fatty acid with the LSM. Both sampling dates and sediment depths were separated along the second axis explaining an additional 3.2% of the variation and correlating mainly with temperature (pseudo‐*F* = 5.0, *p* = .004, contribution = 3.4%).

### 3.2 PLFA ratios

The fungi/bacteria ratio varied significantly with Depth and Zone, but the effect of Zone varied with Season (Table 1). The ratio decreased significantly from 0–5‐cm (0.069 ± 0.031) to 5–10‐cm sediment depth (0.060 ± 0.038). Across the salt marsh zones the ratio was highest in the LSM (0.087 ± 0.042), followed by the PZ (0.062 ± 0.028) and the USM (0.044 ± 0.013), but markedly changed with season with the changes paralleling those of the fungal marker (Figure 2b).

The cy/pre ratio also varied significantly with Depth and Zone, with significant interactions for both Depth and Zone as well as Depth and Season (Table 1). Across the salt marsh zones it generally declined in the order USM (0.277 ± 0.113) > LSM (0.214 ± 0.094)  > PZ (0.122 ± 0.097). This pattern was consistent in the 0–5‐ and 5–10‐cm sediment depths, but the gradient was steeper in 5–10 cm (Figure 3c). Furthermore, the cy/pre ratio was generally higher in the 5–10‐cm than in the 0–5‐cm sediment depth, with the exception of the PZ in July.

The mono/sat ratio varied significantly with Depth, Zone, and Season with significant interactions for both Depth and Zone as well as Zone and Season (Table 1). Generally, it declined from 0–5 (1.506 ± 0.370) to 5–10 cm (1.150 ± 0.310), and from the USM to the LSM to the PZ. In both the USM and PZ, it declined from spring to summer to autumn, but in the LSM it was at a similar level in spring and autumn and lower in summer (Figure 2c). Furthermore, although it generally declined from the USM to the LSM to the PZ, this decline was gradual in the 0–5‐cm depth, whereas in the 5–10‐cm sediment depth, it dropped from the USM to a similarly low level in the LSM and PZ (Figure 3b).

## 4 DISCUSSION

In this study, we aimed to identify changes in the composition and stress indicators of microorganisms across salt marsh zones (USM, LSM, and PZ), seasons (spring, summer, and autumn), and sediment depths (0–5 and 5–10 cm) in the back‐barrier salt marsh of Spiekeroog. Overall, microbial community structure and stress indicators strongly varied among salt marsh zones and between sediment depths, whereas the influence of season was minor.

### 4.1 Salt marsh zones

Generally, plant and bacterial marker concentrations were highest in the USM, algal marker concentration in the PZ, and the fungal marker concentration in the LSM. Soil water content, inundation frequency, salinity, and temperature were identified as major environmental factors driving microbial community composition as reflected by PLFA composition clearly separating the three salt marsh zones.

Plant PLFA marker concentration changed significantly across zones, with high concentration in the USM and LSM, but low concentration in the PZ, confirming our first hypothesis. Consistent with previous studies these results indicate comparatively low plant productivity in the PZ with high loss of detrital material due to tidal movement (Bouchard et al., 1998; Bouchard & Lefeuvre, 2000). Similar to plant PLFA markers, the concentration of bacterial PLFA markers increased from the PZ to the LSM to the USM, again consistent with our first hypothesis. In both sediment depths Gram‐positive bacteria were dominating indicating that mainly complex resources resistant to decomposition such as lignocellulose are processed (Fanin et al., 2019). The presence of litter containing complex compounds such as lignin may have contributed to the dominance of Gram‐positive bacteria in the USM and LSM. Furthermore, the lack of grazing by ungulates at our study site may have contributed to the dominance of Gram‐positive bacteria, as grazing typically increases root exudates favoring Gram‐negative bacteria (Ford et al., 2013). However, in salt marshes Gram‐negative bacteria may also produce lignocellulose‐degrading enzymes (Leadbeater et al., 2021). As predicted by our first hypothesis the cy/pre ratio was highest in the USM. The high soil temperature, lack of inundation, reduced water content, and high salinity of the USM in July 2019 points to frequent evaporation of soil water, resulting in increased salinity (Meier et al., 2020a, 2020b; Meier, Thölen, Lohmus, et al., 2020; Pennings & Bertness, 2001), and this supports our assumption that high temperature and more frequent droughts function as stress for Gram‐negative bacteria. High cy/pre ratio in the LSM and PZ in the upper sediment depth in July and October also point to increased stress for Gram‐negative bacteria in summer and autumn due to high temperature and increased drought. Also supporting our first hypothesis, the mono/sat ratio as an indicator of substrate availability and aerobic microbial activity was highest in the USM. In addition, in the 0–5‐cm sediment depth it increased gradually from the PZ to the LSM to the USM. Contrasting the USM and LSM, in the PZ only small amounts of dead organic matter such as algal wrack was present. In the PZ, both organic matter and bacteria were found to be depleted in ¹³C relative to *Spartina* litter as C4 plant indicating the use of allochthonous marine material (Boschker et al., 1999; Bouillon et al., 2005; Middelburg et al., 1997). In addition, anoxic conditions in the PZ likely contributed to lower bacterial biomass than in the LSM and USM (Buth, 1987; Howarth & Hobbie, 1982).

While overall low, the concentration of the fungal marker 18:2ω6.9 varied significantly across salt marsh zones being highest in the LSM. This was also reflected by the fungi/bacteria ratio, which followed the same pattern, supporting our first hypothesis. The generally low fungal marker concentration suggests that bacteria rather than fungi dominate in salt marsh soil. However, due to their lignocellulose degrading enzymes, fungi may be essential in contributing to the breakdown of salt marsh plant material (Calado & Barata, 2012; Calado et al., 2019). Presumably, fungi only dominate on plant litter material, whereas in soil, bacteria dominate (Benner et al., 1984; Calado et al., 2019; Leadbeater et al., 2021). The review by Calabon et al. (2021) suggests up to 10 fungi genera associated with *Atriplex*, but Wadden Sea vascular plant and fungi associations are understudied. In the PZ, anoxic conditions likely contributed to the low fungal biomass (Calado & Barata, 2012; Calado et al., 2019), as well as displacement of fungi‐bearing litter material. However, the lower fungal marker concentration in the USM than in the LSM remains difficult to explain.

Algal marker concentrations also varied significantly across salt marsh zones. Consistent with our first hypothesis, concentrations were high in the LSM and PZ, and lower in the USM. The presence of the diatom markers 14:0 and 20:5ω3,6,9,12,15, and the green algae marker 16:3ω3 indicates that algae form an important component of the microbial community in each of the three salt marsh zones. Furthermore, the concentration of 20:5ω3,6,9,12,15 in the PZ strongly exceeded that in the LSM and PZ indicating high abundance of diatoms. Given the importance of diatoms in the stabilization of tidal flat sediments (Holland et al., 1974; Hope et al., 2020) and their addition to salt marsh soils with the tide (Redzuan & Underwood, 2020; Scholz & Liebezeit, 2012a), their high abundance in the PZ underlines their key role in frequently inundated salt marsh zones. The reduced presence of 20:5ω3,6,9,12,15 in the LSM and USM likely reflects a shift toward the dominance of heterotrophic microorganisms at higher elevation in the salt marsh. Presumably, also due to high inundation frequency, the flagellate marker 22:6ω3,6,9,12,15,18 only occurred in the PZ. Flagellates are likely to depend heavily on suspended organic marine material (Heijden et al., 2019). However, some of the markers discussed above may have alternative sources including, for example, 14:0, which can also be produced by macroalgae (Biandolino & Prato, 2006; Fleurence et al., 1994; Johnson & Calder, 1973). Furthermore, in terrestrial systems, 14:0 is used as a bacterial marker (Bossio & Scow, 1998; Pollierer et al., 2015). In addition, while diatoms are the dominant producer of 20:5ω3,6,9,12,15,18 (Goutx et al., 2005; Léveillé et al., 1997; Scholz & Liebezeit, 2013; Zhukova & Aizdaicher, 1995), Collembola such as *Folsomia candida* and *Proisotoma minuta* also biosynthesize this marker (Chamberlain et al., 2004, 2006), but this is likely to be of minor importance.

### 4.2 Season

As indicated above, overall, Season only moderately affected PLFA marker concentration and marker ratios. As a main effect, only the algal marker and the mono/sat ratio varied significantly with Season, but the interaction between Season and Zone was also significant for the fungal marker, the fungi/bacteria ratio, and the mono/sat ratio. Furthermore, the interaction between Season and Depth as well as the three factor interaction between Season, Depth, and Zone was significant for the cy/pre ratio.

The fact that the vascular plant marker did not vary significantly with Season disproved our second hypothesis and indicates that the input of vascular plant material to the salt marsh sediments is rather constant in time. Despite the concentration of the bacterial marker not significantly changing with season, the mono/sat ratio varied significantly with season, but the effect differed between zones. In the LSM, the ratio was highest in April, declined in July, and then increased again in October. High loss of detritus in the LSM due to tidal movements (Bouchard et al., 1998; Bouchard & Lefeuvre, 2000) and increased inundation frequency in July 2019 likely contributed to this change, with accumulation of litter contributing to the increase in October (Bossio & Scow, 1998; Meier et al., 2020b). In contrast, in the USM, the ratio was highest in April and steadily declined until October. The high ratio in April suggests an increase in aerobic activity and presence of resources, potentially related to the input of allochthonous organic matter by winter storm tides (Bossio & Scow, 1998; Bouchard et al., 1998; Bouchard & Lefeuvre, 2000). Reductions in the ratio later in the year are likely linked to high salinity and low water content in summer, and increased inundation frequency in October. Overall, changes in the mono/sat ratio appear to be linked to resource input, water content, and salinity suggesting that the response is more complicated than we hypothesized. Changes in the cy/pre ratio with season were complex and depended on both salt marsh zone and depth. In contrast to our second hypothesis, the cy/pre ratio only increased in the LSM and PZ. In July it increased strongly, coinciding with increased inundation frequency of both the LSM and PZ, likely reducing the redox potential of the soil (Bossio & Scow, 1998). In contrast, the cy/pre ratio in the USM remained relatively constant in time, suggesting that Gram‐negative bacteria in the USM remain little affected, pointing to rather constant redox potential conditions across seasons. Overall, contrary to our second hypothesis, the strongest abiotic factors affecting Gram‐negative bacteria appear to be anoxia due to water logging with this being restricted to the LSM and PZ.

Marker PLFAs for algae were the only signal which varied significantly with season as the main factor, confirming our second hypothesis. Concentration across seasons was highest in April and dropped by approximately 15% to a similar level in July and October. This conforms to earlier reports that the biomass of microalgae peaks in spring (Scholz & Liebezeit, 2012b) and that of macroalgae drops in autumn (Kolbe et al., 1995).

The fungal PLFA marker also varied significantly with Season, but the variations differed between salt marsh zones. Contrasting the USM and PZ, in the LSM the fungal marker peaked in July, potentially due to reduced competition with bacteria, but the pattern needs further investigation. In the PZ it was inverse, with the fungal marker being lowest in July and increasing in autumn. Studies on *Spartina* spp. indicate that while leaves remain attached fungi dominate, but once they enter the sediment bacteria take over (Calado & Barata, 2012; Calado et al., 2019; Castro & Freitas, 2000; Newell et al., 1996). The incorporation of plant material heavily colonized by fungi may have contributed to the increased fungal marker in the PZ in autumn. However, bacterial stress indicators (cy/pre and mono/sat) in the PZ in October suggest reduced rather than increased competition with fungi. In contrast, in the USM, the fungal marker concentration was lowest in October, possibly reflecting a declining input of plant residues into the soil of the USM until autumn. Generally, the fungi/bacteria ratio followed the seasonal pattern of the fungal marker suggesting that seasonal dynamics were driven by changes in fungal rather than bacterial biomass. However, as a note of caution, we did not fully capture seasonal variations as we did not sample in winter and, therefore, may have missed variations due to low temperature and winter storms.

### 4.3 Sediment depth

Sediment depth was the strongest factor affecting microbial community composition of the studied salt marsh soils. Most of the PLFA markers were approximately halved in the 5–10‐cm compared to the 0–5‐cm sediment depth, and bacteria became more dominant deeper in soil, as indicated by the fungi/bacteria ratio. Overall, this supports our third hypothesis that due to reduced resource availability and redox potential the biomass and activity of microorganisms are declining with sediment depth.

The reduction in the bacteria marker concentration by about 50% in the 5–10‐cm compared to the 0–5‐cm sediment depth across salt marsh zones suggests that plant roots only play a minor role in oxygenating deeper sediment depths (Armstrong et al., 1985; Buth, 1987). The stress indicators mono/sat and cy/pre ratios also reflect increased oxygen limitation in deeper soil, supporting our third hypothesis. Across sediment depths both indicators were generally highest in the USM followed by the LSM and PZ, presumably reflecting the general decline in oxygenation at lower salt marsh zones. However, the decrease was least pronounced in the PZ, suggesting reduced impact of oxygen stress. This agrees with previous indications of anaerobic bacteria dominance and anaerobic breakdown of detritus in the PZ (Bossio & Scow, 1998; Howarth & Hobbie, 1982; Wixon & Balser, 2013). In the upper sediment depth the cy/pre ratio increased during the year in the LSM and PZ, particularly between April and July, whereas it remained constant in the USM. The changes in the PZ and LSM coincide with a stark increase in inundation frequency in these salt marsh zones in 2019 (Meier et al., 2020b), presumably resulting in waterlogging of the upper sediment depth reducing the redox potential.

The decrease in the fungal marker concentration with sediment depth presumably reflects both reduced oxygen and plant resource availability deeper in soil (Calado & Barata, 2012; Padgett et al., 1986, 1989). The decrease in fungi with sediment depth was also reflected in the decrease in the fungi/bacteria ratio indicating that the decline in fungi is more pronounced than that of bacteria.

Algal marker concentrations also declined with sediment depth, presumably reflecting light limitation. Among the algal PLFA markers, the reduction was strongest for the diatom marker 20:5ω3,6,9,12,15, which was reduced by 95% (PZ), 100% (LSM), and 97% (USM) in the 5–10‐cm compared to the 0–5‐cm sediment depth. Epipelic diatoms may vertically migrate to the surface of the soil during low tide to photosynthesize (Cartaxana et al., 2016; Redzuan & Milow, 2019), thereby actively avoiding burial. In contrast, the green algae marker 16:3ω3,6,9 was only reduced by 40% (PZ), 47% (LSM) and 28% (USM) in the 5–10‐cm compared to the 0–5‐cm sediment depth. The fact that on average algal marker concentrations in the 5–10‐cm depth still were about half of that in the 0–5‐cm sediment depth presumably reflects the burial of green algal material in marine sediment due to tidal forces (Buchan et al., 2003; Currin et al., 1995). Buried organic matter probably decays slowly as has been shown for *S*. *anglica* roots (Hemminga et al., 1988), which is likely related to reduced decomposition of complex plant compounds such as lignocellulose at low oxygen conditions deeper in the sediment (Howarth & Hobbie, 1982). However, as discussed previously, some of these marker PLFAs may have alternative sources, which requires further investigation.

## 5 CONCLUSIONS

Overall, this study provided insight into the spatial and temporal dynamics of the structure and functioning of the soil microbial community in salt marsh soils. The results highlight strong variations among salt marsh zones and with sediment depth, both related to variations in abiotic conditions, in particular, not only inundation frequency‐associated water logging and salinity but also temperature. In contrast, seasonal variations are much less pronounced and limited to algae and indicators of bacterial substrate availability (mono/sat ratio) as well as fungi and the fungi/bacteria ratio, but the latter depended on salt marsh zone. Changes in algae markers among seasons reflect changes in inundation frequency. The overall stability in PLFA patterns in time indicate little influence of temperature on the microbial communities. Instead, spatial dynamics were the strongest factor—both across vertical (sediment depth) and horizontal scales (salt marsh zones)—indicating a microbial community regulated by inundation frequency and the associated abiotic conditions including water content, salinity, and oxygen availability. These findings give insights into the dynamics of microbial communities of Wadden Sea salt marshes and associated functions, which is urgently needed in the face of global change and the potential of Wadden Sea salt marshes for blue carbon sequestration.

## CONFLICT OF INTEREST

The authors declare no conflicts of interests in this study.

## AUTHOR CONTRIBUTIONS

**Maria Rinke:** Conceptualization (equal); Data curation (lead); Formal analysis (equal); Investigation (equal); Methodology (equal); Validation (equal); Visualization (lead); Writing – original draft (lead); Writing – review & editing (equal). **Mark Maraun:** Conceptualization (equal); Formal analysis (supporting); Funding acquisition (lead); Investigation (supporting); Resources (equal); Supervision (equal); Visualization (supporting); Writing – original draft (supporting); Writing – review & editing (supporting). **Stefan Scheu:** Conceptualization (equal); Formal analysis (supporting); Funding acquisition (lead); Resources (lead); Supervision (lead); Visualization (equal); Writing – original draft (supporting); Writing – review & editing (lead).

## Supporting information

Supplementary Material

## Data Availability

The data can be accessed on DRYAD (https://doi.org/10.5061/dryad.95x69p8n6).
